# Effectiveness of Saikokaryukotsuboreito (Herbal Medicine) for Antipsychotic-Induced Sexual Dysfunction in Male Patients with Schizophrenia: A Description of Two Cases

**DOI:** 10.1155/2014/784671

**Published:** 2014-01-27

**Authors:** Tsuboi Takashi, Hiroyuki Uchida, Takefumi Suzuki, Masaru Mimura

**Affiliations:** ^1^Department of Neuropsychiatry, Keio University School of Medicine, 35 Shinanomachi, Shinjuku-ku, Tokyo 160-8582, Japan; ^2^Geriatric Mental Health Program, Centre for Addiction and Mental Health, Toronto, ON, Canada M5T 1R8; ^3^Department of Psychiatry, Inokashira Hospital, Tokyo 181-0012, Japan

## Abstract

Antipsychotics sometimes cause sexual dysfunction in people with schizophrenia. The authors report the effectiveness of Saikokaryukotsuboreito (Japanese traditional herbal medicine, Chai-Hu-Jia-Long-Gu-Mu-Li-Tang in Chinese) for antipsychotic-induced sexual dysfunction in two male patients with schizophrenia. The first patient was a 28-year-old man with schizophrenia who suffered erectile dysfunction induced by olanzapine 10 mg/day; the erectile dysfunction significantly improved following the treatment of Saikokaryukotsuboreito 7.5 g/day. The other case was a 43-year-old man with schizophrenia who was receiving fluphenazine decanoate at 50 mg/month and had difficulties in ejaculation; add-on of Saikokaryukotsuboreito 7.5 g/day recovered his ejaculatory function. There has been no report on the effectiveness of Japanese herbal medicine formulations for antipsychotic-induced sexual dysfunction. Although the effectiveness of Saikokaryukotsuboreito needs to be tested in systematic clinical trials, this herbal medicine may be a treatment option to consider for this annoying side effect.

## 1. Introduction

Antipsychotic drugs sometimes cause sexual dysfunction in patients with schizophrenia; the incidence rate has been reported to be as high as 50% in male patients [1]. Among sexual side effects, disturbances of erection and ejaculation are frequent with reported incidence rates being as high as 30–60% [2]. These adverse events can cause substantial subjective burden and are expected to result in an impaired quality of life, potentially leading to poor adherence to antipsychotic treatment [3].

Kampo, or Japanese traditional herbal medicine, has been used in Japan for more than 1300 years as an alternative treatment or sometimes combined adjunctively with the Western modern medicine. Today, 148 Kampo formulations have been approved for use in clinical practice by the Japanese Ministry of Health, Labour and Welfare; some of them have been reported to improve psychotropic side effects as well as psychiatric symptoms [4, 5].

Here we report on two male patients with schizophrenia in whom a Kampo prescription, Saikokaryukotsuboreito (Chai-Hu-Jia-Long-Gu-Mu-Li-Tang in Chinese), which has often been used for sexual dysfunction in general, successfully diminished antipsychotic-induced sexual dysfunction.

## 2. Case Presentation

### 2.1. Case 1

A 28-year-old single man who did not have any past history of psychiatric or physical illnesses visited our hospital because of psychotic symptoms characterized with delusions, conceptual disorganization, and hallucinations. He was diagnosed with schizophrenia according to the Diagnostic and Statistical Manual of Mental Disorders, fourth edition (DSM-IV) and then treated with risperidone 3 mg/day. His psychotic symptoms improved in approximately two months, but he started to suffer from erectile dysfunction that he had not experienced before the treatment. Risperidone was switched to olanzapine 10 mg/day. Six months later, while he achieved a state of remission with the same dose of olanzapine, erectile dysfunction still continued. Routine hematological laboratory results were within the reference ranges. He was referred to an urologist with no remarkable findings. A score in the International Index of Erectile Function (IIEF-5) [6] was 10 out of 25, indicating a moderate severity of erectile dysfunction. Saikokaryukotsuboreito was then concomitantly prescribed at 2.5 g t.i.d. Two months later, erectile dysfunction significantly improved and the score in the IIEF-5 increased to 21, indicating that erectile function returned to almost normal. He appears to be adherent to medications throughout, and has maintained remission on the same regimen without any side effects until now. No remarkable changes in mood or anxiety were noted as a result of adding Saikokaryukotsuboreito to olanzapine.

### 2.2. Case 2

A 43-year-old married man with a 12-year history of schizophrenia (DSM-IV) had poor medication adherence and frequently relapsed. The use of fluphenazine decanoate at 50 mg/month stabilized his psychiatric conditions; however, he started to complain of difficulties in ejaculation although he could erect with an IIEF-5 score of 21. He previously experienced the same problem when he was treated with haloperidol or olanzapine as monotherapy but never had that before treatment with antipsychotics. Urological examinations failed to identify any organic abnormalities such as retrograde ejaculation. Moreover, he had no past history or current physical illnesses. In addition, there was no abnormal finding in the brain MRI scan as well as routine laboratory tests. Saikokaryukotsuboreito was started at 2.5 g t.i.d. in addition to fluphenazine decanoate. Six weeks later, ejaculatory function was restored and he was able to obtain sexual pleasure by ejaculation. However, eleven weeks later, ejaculatory dysfunction recurred when he voluntarily stopped taking the medication. His sexual function recovered again by resuming the same dose of Saikokaryukotsuboreito, and no remarkable changes in mood or anxiety were noted throughout with adjunctive use of this Kampo formulation.

## 3. Discussion

These two cases indicated that antipsychotic-induced difficulties in erection and ejaculation were ameliorated by the concomitant administration of Saikokaryukotsuboreito at a daily dose of 7.5 g with ongoing antipsychotic treatment. Furthermore, in one instance, ejaculatory dysfunction recurred after the medication was voluntarily stopped, which was recovered again by resuming the same dose of Saikokaryukotsuboreito, and this provides support for the efficacy of this adjunctive treatment.

Sexual dysfunction is a common problem in schizophrenia. This symptom can be caused by a variety of conditions such as a symptom of schizophrenia itself, physical complications, and the use of antipsychotics. In the two cases that we presented herein, the sexual dysfunction was expected to be an antipsychotic-induced side effect in light of the time-effect relationship. In fact, neither of the two patients suffered from sexual dysfunction before starting the current antipsychotic drugs. It may have been an option to reduce the dose or switch antipsychotic drugs in these patients. However, in case 1, the dose was already low; in addition, the patient did not wish to change antipsychotic drugs since it was effective; this is a situation commonly encountered in actual clinical practice. In case 2, the dose of fluphenazine decanoate was already modest, and it was difficult to switch drugs because this depot antipsychotic effectively stabilized his symptoms.

Besides antipsychotic dose reduction and switching, several drugs have been shown to be effective for the management of sexual dysfunction in male patients receiving antipsychotic medications [7]. Phosphodiesterase type 5 (PDE-5) inhibitors have been often used to reverse sexual dysfunction induced by antipsychotics; in fact, sildenafil has been found to be effective [8, 9]. However, its use is still limited because of its high cost and physical contraindications. Yohimbine, which is an alkaloid with stimulant and aphrodisiac effects, has also been shown to be effective in the treatment of male impotence [10, 11]. This medication is however not recommended for anyone with a psychiatric disorder since it could cause emotional disturbances such as anxiety, irritability, and nervousness. Evidence on adjunct selegiline is also weak [8], highlighting a difficult-to-manage situation with few viable treatment options.

To our knowledge, there has been no report on the effectiveness of Kampo formulations for treating sexual dysfunction induced by antipsychotics. In fact, while a PubMed search (last search: September 2013) with keywords of “antipsychoticsm,” “schizophrenia,” and “sexual dysfunction” yielded 206 hits, adding the term “herbal” or “kampo” reduced the number of hits to zero. Adding the term “adjunctive” resulted in five hits, and it was found that a comprehensive literature review on this topic failed to evaluate adjunctive treatment with herbal medications against antipsychotic-induced sexual disturbances [8]. On the other hand, in animal studies, Saikokaryukotsuboreito is reported to suppress excitotoxicity of glutamate [12] and to attenuate increase in monoamine levels induced by emotional stress [13]. Further, it may have some anxiolytic [14] or antidepressive [15] effects. Few studies on this medication were performed in humans but a positive effect of this medication has been reported among eugonadal men with late-onset (i.e., over 40 years of age) hypogonadism-related symptoms [16].

Saikokaryukotsuboreito (a daily dose of 7.5 g) consists of Bupleurum Root (5.0 g), Pinellia Tuber (4.0 g), Cinnamon Bark (3.0 g), Poria Sclerotium (3.0 g), Scutellaria Root (2.5 g), Jujube, Ginseng (2.5 g), Oyster Shell (2.5 g), Longgu (2.5 g), and Ginger (1.0 g). Saikokaryukotsuboreito is indicated for a relief of hypertension, arteriosclerosis, chronic renal disease, neurasthenia, neurotic palpitations, epilepsy, hysteria, and erectile dysfunction [16]. Furthermore, Saikokaryukotsuboreito has been found to be effective for hypogonadism-related symptoms such as erectile dysfunction in eugonadal patients older than 40 regardless of serum concentration of testosterone [16]. Although precise mechanisms of action in ameliorating sexually dysfunction remain unknown, the following potential mechanisms may be involved. Firstly, saponins, which are glycosides with a distinctive foaming characteristic and can be extracted from Saikokaryukotsuboreito, have been shown to be effective in diminishing erectile dysfunction [17–19]. For example, Korean Red Ginseng that includes saponins improves erectile function [18]. Another possibility is that Saikokaryukotsuboreito could improve sexual dysfunction through inhibition of PDE-5. In fact, a health supplement extracted from fresh oyster has been reported to show some activity as a PDE-5 inhibitor [20]; Saikokaryukotsuboreito that includes oyster shells may share the same clinical property.

While the exact mechanisms of action remain to be elucidated, more work is also indicated to counteract this common but difficult-to-treat side effect. Furthermore, good effectiveness and tolerability that we observed in our patients need to be confirmed in larger systematic clinical trials. In addition, effectiveness in female patients remains an issue to be addressed.

In conclusion, the concomitant use of Saikokaryukotsuboreito diminished antipsychotic-induced dysfunction in erection and ejaculation in two men with schizophrenia. Although the effectiveness of Saikokaryukotsuboreito needs to be tested in systematic clinical trials, this herbal medicine may be a treatment option to consider against this annoying side effect.
